# In Vitro Antioxidant Activity, Bioaccessibility, and Thermal Stability of Encapsulated Strawberry Fruit (*Fragaria × ananassa*) Polyphenols

**DOI:** 10.3390/foods12214045

**Published:** 2023-11-06

**Authors:** Faith Seke, Oladipupo Q. Adiamo, Yasmina Sultanbawa, Dharini Sivakumar

**Affiliations:** 1 Phytochemical Food Network Group, Department of Crop Sciences, Tshwane University of Technology, Pretoria West, Pretoria 0001, South Africa; sekef@tut.ac.za; 2 Australian Research Council Industrial Transformation Training Centre for Uniquely, Australian Foods, Queensland Alliance for Agriculture and Food Innovation, Centre for Food Science and Nutrition, The University of Queensland, Indooroopilly, QLD 4068, Australia; o.adiamo@uq.edu.au (O.Q.A.); y.sultanbawa@uq.edu.au (Y.S.)

**Keywords:** anthocyanins, biopolymers, antioxidant activity, simulated gastrointestinal digestion, phytochemicals, mucilage

## Abstract

Bioactive compounds in red fruits, such as strawberries, are vulnerable to digestion, and encapsulation has become an alternative for their protection. This study aims at encapsulating strawberry juice (SJ) by freeze-drying with pea protein and okra mucilage (SJPO), pea protein and psyllium mucilage (SJPP), and pea protein, psyllium mucilage, and okra mucilage (SJPPO) and investigating the in vitro release. The highest encapsulation efficiency was observed in capsule SJPPO (95.38%) and the lowest efficiency in SJPO (82.45%). Scanning electron microscopy revealed an amorphous glassy structure for the structure of the strawberry microcapsules, and X-ray diffraction confirmed that observation. However, X-ray diffraction further showed that SJPPO was crystalline, indicating a tighter crosslinking density than the other microcapsules. Fourier transform infrared spectroscopy showed peaks at 3390 and 1650 cm^−1^, confirming the presence of polyphenols and polysaccharides in the strawberry microcapsules. Thermal stability was higher for SJPPO, and the observed thermal transitions were due to the bonds formed between the polymers and polyphenols. Pelargonidin 3-glucoside, cyanidin 3-glucoside, cyanidin, delphinidin, malvidin 3-glucoside, ellagic acid, chlorogenic acid, catechin, and kaempferol were identified in the strawberry microcapsules. Digestion affected the compounds’ content; the bioaccessibility for SJ was 39.26% and 45.43% for TPC and TAC, respectively. However, encapsulation improved the bioaccessibility of both TPC (SJPP, 51.54%; SJPO, 48.52%; and SJPPO, 54.39%) and TAC (SJPP, 61.08%; SJPO, 55.03%; and SJPPO, 71.93%). Thus, encapsulating pea protein isolate, psyllium mucilage, and okra mucilage is an effective method to facilitate targeted release and preserve the biological activities of fruits.

## 1. Introduction

One of the significant and consistent recommendations for global human health is to increase the consumption of fruits and vegetables up to at least five (portions) a day [1]. The distinct red colour present in strawberries has been attributed to the presence of anthocyanins (cyanidin 3-glucoside, pelargonidin 3-glucoside, cyanidin 3-rutinoside pelargonidin 3-rutinoside, and pelargonidin 3-arabinoside). Strawberries also contain other polyphenolic compounds, such as protocatechuic acid, catechin, p-coumaric acid, and ellagic acid [2]. The broad spectrum of health benefits of these polyphenolic compounds has led epidemiological studies to suggest a link between their consumption and the prevention of several diseases [3]. However, identifying polyphenolic compounds may not accurately predict their impact in vivo. The impact of bioactive compounds on health is influenced by a number of factors, including food processing, changes that take place during digestion, absorption, and cellular metabolism [4]. The bioactivity of polyphenolic compounds after digestion depends on their bioaccessibility and bioavailability. In order to evaluate the nutritional value of the bioactive component and determine the quantities needed to satisfy nutritional requirements, it is essential to understand bioaccessibility [5]. Natal plum anthocyanins and phenolic compounds were highly unstable and had minimal bioaccessibility during simulated gastrointestinal digestion [6]. In addition, Herrera-Balandrano et al. [7] reported that the changes in digestion conditions altered the bioaccessibility of the anthocyanins in rabbiteye blueberry fruit during digestion. Therefore, the instability of polyphenols during gastrointestinal digestion and their limited bioaccessibility may limit their activity and therapeutic benefits [6]. Research has shown that only 8% of dietary flavonoids and 10% of nutritional polyphenols are absorbed in the gastrointestinal tract; the rest are broken down by the colonic microbiota in the colon [8]. As a result, the food industry is continuously seeking practical and cost-effective ways to preserve the bioactive properties of polyphenols. Microencapsulation is a suggested strategy to protect bioactive substances during gastrointestinal digestion, potentially increasing their bioaccessibility [9]. The choice of wall material and encapsulation technique affects the chemical and physical characteristics of the microparticles in an interdependent manner [10]. 

The combination of hydrocolloids and protein isolates has drawn much interest because of the encouraging outcomes [10]. Combining materials with various qualities results in better protection and appropriate release properties [9]. In addition to being a bioadhesive, binder, film coating, and suspending agent, okra mucilage is a low-cost hydrocolloid [11]. Psyllium mucilage not only helps maintain digestive health and promotes the development of probiotic bacteria but also functions as an efficient polyphenol wall material [12]. Alginate/psyllium mucilage beads have effectively enhanced stability and in vitro anthocyanin release in different research [13]. It has been shown that psyllium improves the stability of components encapsulated in acidic products, such as yogurt [14]. Compared to sodium alginate alone, okra mucilage [15] has been shown to enhance the stability of encapsulated probiotics in microspheres during storage. It was noted that these dietary fibres and health-promoting oils might be effectively integrated by encasing polyunsaturated fenugreek oil in okra or psyllium mucilage copolymers [16]. Further investigation is required to determine the viability and efficacy of mixing mucilage and protein isolates to expand the range of affordable possibilities. The strawberry flavour was previously encapsulated by spray drying and freeze-drying with a wall material blend comprising maltodextrin, soluble fibre, modified starch, cyclodextrin, and Arabic gum [17]. In that study, strawberry microcapsules produced by spray drying had a more uniform and spherical structure, and microcapsules made by freeze drying recovered more aroma-active compounds, although it was recommended that spray drying was more widely applicable. Gong et al. [18] employed spray drying of strawberry puree with combinations of maltodextrin (MD) and whey protein isolated (WPI). Results revealed that MD–WPI boosted the efficiency of powder recovery up to 56% but resulted in microparticles with shrunken surfaces. Similarly, Leyva-Porras et al. [19] noticed rough surfaces and deflated balls after drying strawberry juice with MD and powders. Another study found that using spray-dried powders of *A. angustifolia* fructans as an encapsulating agent for strawberry juice can prevent the intake of significant amounts of refined carbohydrates normally employed in this procedure, such as maltodextrins [20]. These studies on strawberries suggest the feasibility of encapsulating strawberry flavours and bioactive compounds. 

However, there is limited information on the effects of encapsulation on the bioaccessibility of strawberry bioactive compounds, specifically anthocyanins. The availability of this information would assist in broadening consumer awareness of the consumption of encapsulated strawberry powder and its benefits. In order to broaden the low-cost options, more research is needed to understand the feasibility and effectiveness of combining mucilage and protein isolates. Thus, in light of the above-mentioned, the objective of this study was to investigate the effects of combining pea protein isolate with okra mucilage or psyllium mucilage on strawberry encapsulation efficiency, phenolic and anthocyanin content, bioaccessibility of individual polyphenols, and antioxidant activity during in vitro digestion.

## 2. Materials and Methods

### 2.1. Chemicals and Fruits

Strawberries were purchased at a fruits and vegetable market in Pretoria, South Africa. The chemicals were all purchased from Sigma Aldrich Co. (Johannesburg, Gauteng, South Africa). Chemical reagents, standards, and solvents used in this experiment were analytical grade.

### 2.2. Okra and Psyllium Mucilage Extraction

Psyllium husk mucilage was extracted by combining husks with water in a 1:100 (*w*/*v*) ratio for 90 min at 80 °C with continual agitation. A previously published precipitation technique [21] was used to separate okra mucilage (OM). The obtained mucilage was freeze-dried using a Benchtop Freeze Dryer at −47 to −53 °C (VirTis Sp Scientific, Model # 2kBTES-55, Gardiner, NY, USA) and stored at 4 °C until use.

### 2.3. The Development of the Strawberry Juices

The development of encapsulated strawberry juices in different biopolymers was conducted following a method described by Seke et al. [22] with some modifications. Four different formulations were distinctively developed for strawberry by blending the individual juices with pea protein isolate (1.5%) and psyllium mucilage (3%) (SJPP), pea protein isolate (1.5%) and okra mucilage (3%) (SJPO), pea protein isolate (1.5%), psyllium mucilage (1.5%) and okra mucilage (1.5%) (SJPPO), and a control (SJ) without any biopolymers (Supplementary Table S1). The resulting strawberry juices were freeze-dried after 48 h of freezing. The resultant microcapsules were kept at 4 °C in brown bottles.

### 2.4. Microcapsule Characterization

#### 2.4.1. Microcapsule Microstructure—Scanning Electron Microscopy (SEM)

The freeze-dried strawberry microcapsules’ microstructure was investigated using a technique outlined by Seke et al. [13]. The samples were viewed at a magnitude of 2000, 2500, 5000, and 10,000. 

#### 2.4.2. Thermal Analysis—TGA

Mendes et al. [23] proposed a technique for thermogravimetric analysis (TGA). The temperature range was set up to 900 °C and a heat rate of 10 °C/min in nitrogen. 

#### 2.4.3. X-ray Diffraction (XRD)

X-ray diffractograms of the microcapsules were obtained using a Shimadzu model LabX XRD-6000 diffractometer with a scan range of 4.5–80° (2θ) [24].

#### 2.4.4. FT-IR Spectroscopy

In accordance with the protocol put out by Seke et al. [13], the interaction between polymers and polyphenols in the freeze-dried strawberry microcapsules was examined using Fourier transform infrared spectroscopy (FT-IR). With a spectra resolution of 4 cm^−^^1^, the infrared spectra were within the wavelength range of 600–4000 cm^−^^1^.

### 2.5. In Vitro Digestion of Strawberry Juices

Using a technique outlined by Seke et al. [22], in vitro digestion of the unencapsulated and encapsulated strawberry juice powder was carried out by subjecting the encapsulated powder samples to simulated gastric juice at pH 2.5 and incubating for 120 min, followed by intestinal fluid at pH 7.5 for an additional 120 min (Supplementary Figure S1). After the process, the collected samples were stored at −80 °C, including an enzyme-containing digestion blank without the samples. Equation (1) was used to calculate the bioaccessibility (what is left after intestinal digestion) using the following:

(1) $$\mathrm{Bioaccessibility}\%=\frac{BSI}{BND}\times 100$$

The phenolic concentration after the intestinal phase is denoted by *BSI* (mg/100 g), while the phenolic content in the undigested sample is denoted by *BND* (mg/100 g).

### 2.6. Extraction of Phenolic Compounds in Strawberry Juices Powder

Phenolic extraction was conducted using the method described by Seke et al. [22]. The phenolic compounds from the samples (100 mg) were extracted using 80% methanol with 1% formic acid. The samples were ultrasonicated for 35 min at 37 °C, then centrifuged at 1000× *g* for 30 min at 4 °C (Hermle Z326k, Hermle Labortechnik GmbH, Wehingen, Germany). The extracts were then stored at −80 °C until further analysis.

### 2.7. Total Phenolic Content (TPC)

TPC was determined using the Folin–Ciocalteu method described by Seke et al. [6]. The absorbance was measured at 750 nm, and the standard used was gallic acid. The results were presented as mg GAE/100 g DW (gallic acid equivalent).

### 2.8. Total Anthocyanin Content (TAC)

The pH difference was used to calculate the total anthocyanin content using a technique described by Seke et al. [13]. Two distinct buffers, pH = 1.0 and pH = 4.5, were used to test the samples’ absorbance at 510 and 700 nm. The findings were presented as milligrams of cyanidin 3-glucoside (Cyd-3-Glu) per 100^−^^1^ g dry powder weight. To determine the encapsulation efficiency, Equation (2) was used.

(2) $$Encapsulation\;efficiency\;\%=\frac{Anthocyanin\;content\;in\;the\;encapsulated\;samples}{Anthocyanin\;content\;in\;the\;extract}\times 100$$

### 2.9. Quantification of Targeted Anthocyanin Components

Using a technique outlined by Seke et al. [6], the anthocyanins in strawberry juice were measured using HPLC-DAD (Model Flexar TM 89173-556 (PerkinElmer, Waltham, MA, USA). The regression equations, LODs, and LOQs are outlined in Supplementary Table S2. For phenolics and anthocyanins, chromatograms were generated at wavelengths of 320 nm and 520 nm, respectively. Supplementary Figure S2A–C display a selection of these chromatograms.

### 2.10. Antioxidant Capacity

The techniques outlined by Seke et al. [6] were used to assess the antioxidant content of the strawberry juices, both encapsulated and unencapsulated. The ferric reduction antioxidant potential (FRAP), which was measured at 593 nm, the absorbances for the DPPH assay, which was measured at 517 nm, and the radical scavenging capacity of ABTS, which was measured at 734 nm, were all determined using spectrometry techniques. The results for both DPPH and ABTS were expressed as IC_50_ (mg mL^−1^), and the results for FRAP were expressed as Trolox equivalent antioxidant activity (mM TEAC/g).

### 2.11. Statistical Analysis

To ensure the accuracy of the results, the experiments were conducted at least three times in triplicate or quadruplicate. The data were expressed using the mean and standard error of the mean. The one-way analysis of variance (one-way ANOVA) was used along with Tukey’s multiple comparison test to determine whether there were any significant differences between the means of the experimental groups. Additionally, the Pearson correlation analysis was used to establish the correlation between total phenolic content, total anthocyanin content, anthocyanin compounds, and antioxidant activities.

## 3. Results and Discussion

### 3.1. Anthocyanin Encapsulation Efficiency of Encapsulated Strawberry Juice

**Figure 1:**
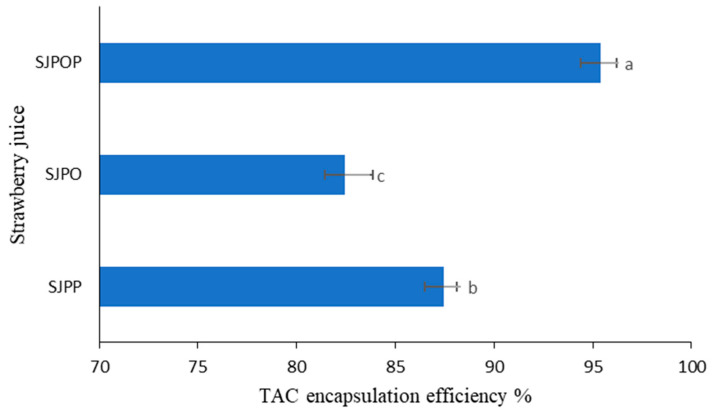
Anthocyanin encapsulation efficiency of encapsulated of strawberry (*Fragaria × ananassa*) juice. Error bars show the standard deviation value; bars with different letters are significantly different, *p* < 0.05. SJPP: Strawberry (*Fragaria × ananassa*) juice + pea protein isolate + psyllium mucilage; SJPO: Strawberry (*Fragaria × ananassa*) juice + pea protein isolate + okra mucilage; SJPPO: Strawberry (*Fragaria × ananassa*) juice + pea protein isolate + okra mucilage + psyllium mucilage.

The success of anthocyanin encapsulation in microparticles can be determined by its encapsulation efficiency, which minimizes surface anthocyanin content and maximizes active compound retention [25]. Figure 1 illustrates the percentage of total anthocyanin retained within the matrix of the strawberry samples after encapsulation.

The results indicate that the coating used for encapsulation plays a crucial role in retaining anthocyanin compounds within the matrix. Pea protein isolate/okra mucilage/psyllium mucilage was found to be the most effective wall material for strawberry extracts, retaining 95.38% of anthocyanin compounds. The lowest encapsulation efficiency (82.45%) was observed in the strawberry extracts encapsulated using pea protein isolate/okra mucilage as the wall material. Encapsulation efficiency can be improved by adding various types of polymers to our pea protein system. In addition, the interaction between added polysaccharides and hydroxyls in strawberry extract facilitates hydrogen bonding and linkage with accessible carboxylic acid groupings on utilized derivatives. Psyllium mucilage’s protein moiety was also proposed to enhance the encapsulation efficiency of Natal plum polyphenols [22].

The attachment of protein molecules to carbohydrate chains creates high-quality films that improve molecule encapsulation and increase the stability of anthocyanins by making them less susceptible to nucleophilic attacks [26]. One possible explanation for the behaviour of anthocyanins is the interaction between their flavylium cation and dextrin, which results in the formation of a complex nature that prevents them from transforming into an unstable state [27]. The changes in morphology can partly explain this behaviour during the drying process [13]. Also, the composition of the solvent can strongly affect the polyphenols and the extractability from different matrices, and therefore on the results for encapsulation efficiency.

### 3.2. Impact of Encapsulation on the Surface Morphology and X-ray Diffraction Analysis of the Strawberry Microcapsules

**Figure 2:**
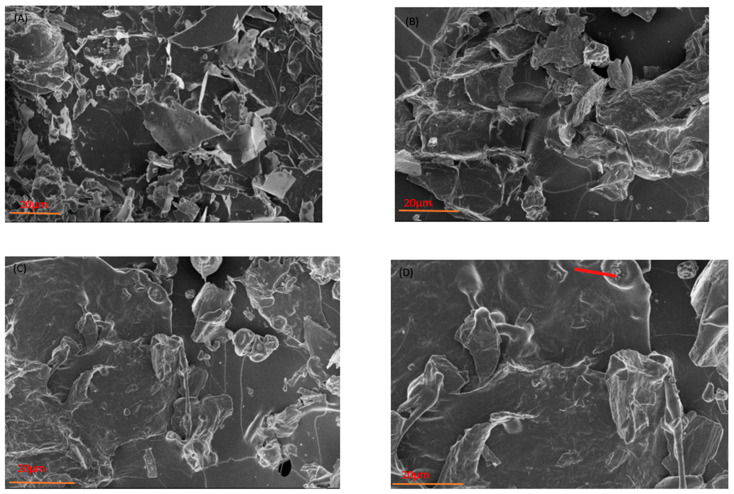
SEM microphotographs of strawberry microcapsules. (**A**) SJ: strawberry juice, (**B**) SJPO: strawberry (*Fragaria × ananassa*) juice + pea protein isolate + okra mucilage, (**C**) SJPP: strawberry (*Fragaria × ananassa*) juice + pea protein isolate + psyllium mucilage, (**D**) SJPPO: strawberry (*Fragaria × ananassa*) juice + pea protein isolate + okra mucilage + psyllium mucilage.

The SEM images in Figure 2 show the morphologies of powdered anthocyanins that have been enclosed. All four samples displayed uneven shrinkage and rough surfaces due to using the best formulas. However, the particles possessed amorphous glassy forms, typical of freeze-dried solid materials that have been crushed and dehydrated separately.

The anthocyanins that are trapped can be shielded from heat and oxidation by the glassy structures. The SJPPO microcapsules were smaller, more uniform, spherical particles that aggregated together, perhaps because they were sticky, confirming their crystalline nature.

However, the SJPP and SJPPO micrographs showed little to no cracks, indicating that the powders were oxidation-resistant. A previous study on the effect of psyllium-based wall material on the encapsulation of Natal plum anthocyanins reported a clear and smooth surface [13]. Furthermore, no loose cells were seen on the surfaces of the microcapsule particles, demonstrating that freeze-drying successfully produced microcapsules from all the utilized carrier agents. To study the crystal lattice arrangement and crystallinity characteristics of the anthocyanin-packed microparticles, X-ray diffraction analysis (XRD) was essential.

**Figure 3:**
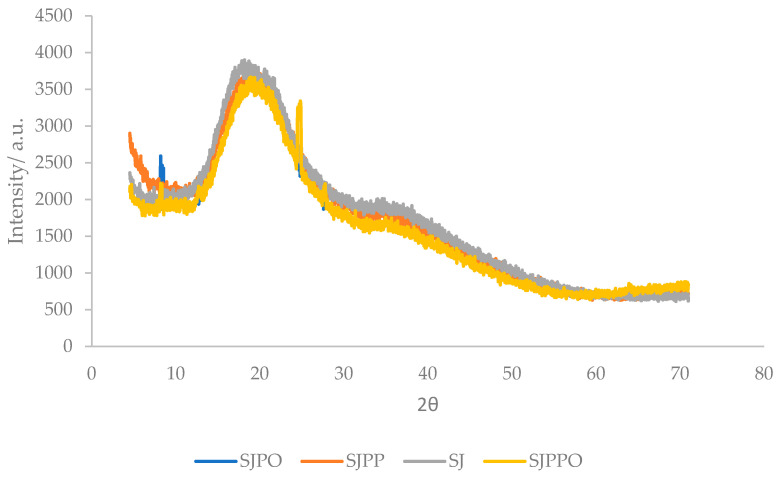
XRD analysis of strawberry microcapsules. SJ: strawberry juice, SJPO: strawberry (*Fragaria × ananassa*) juice + pea protein isolate + okra mucilage, SJPP: strawberry (*Fragaria × ananassa*) juice + pea protein isolate + psyllium mucilage, SJPPO: strawberry (*Fragaria × ananassa*) juice + pea protein isolate + okra mucilage + psyllium mucilage.

Figure 3 displays the XRD analysis outcomes for the powders that were encapsulated. These graphs show that, regardless of the formulations employed, all the samples were in the amorphous phase since there was only one wide peak that was accompanied by several sounds. This outcome was in line with the SEM morphologies, which showed that all the samples had amorphous forms. According to Ramrez-Ambrosi et al. [28], the type of wall materials utilized had no impact on the crystallinity of the powders that were enclosed. Amorphous samples, on the other hand, are very hygroscopic and absorb water while in storage, which might lead to nutritional degradation, collapse of the microstructure, and probable microbial growth [29,30].

On the other hand, SJPPO was more tightly crosslinked than the other microcapsules, indicating that it was crystallized as seen by the sharp, defined peaks in a highly ordered form.

### 3.3. Fourier Transform Infrared Spectroscopy (FTIR) Analysis

**Figure 4:**
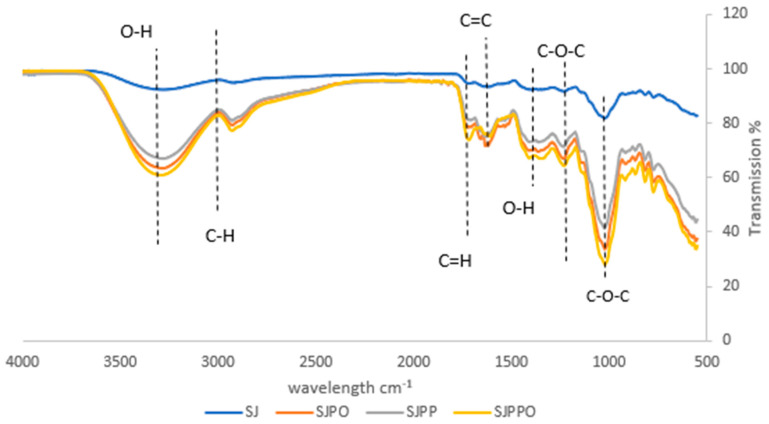
Fourier transform infrared spectroscopy measurements (FT-IR) of strawberry microcapsules. SJ: strawberry juice, SJPO: strawberry (*Fragaria × ananassa*) juice + pea protein isolate + okra mucilage, SJPP: strawberry (*Fragaria × ananassa*) juice + pea protein isolate + psyllium mucilage, SJPPO: strawberry (*Fragaria × ananassa*) juice + pea protein isolate + okra mucilage + psyllium mucilage.

The presence of distinct peaks at around 3390, 2892, 1700, 1650, 1487, 1352, and 1007 cm^−^^1^ demonstrated that anthocyanins were the main component of the extract in the FTIR analysis (Figure 4). The broad peak at 3390 cm^−^^1^ suggested that O-H bonds were vibratingly stretching. Free hydroxyl groups, intramolecular and intermolecular bonding, and other factors might cause this. The distinctive bands between 1800 cm^−1^ and 1000 cm^−1^ that make up the so-called fingerprint region of the spectrum were responsible for polysaccharides [31]. The presence of proteins in the microcapsules was confirmed by the amide I band at 1627 cm^−1^, which corresponds to the C=O stretch vibrations of peptide connections. The bending vibration of C-O-C groups detected carbohydrates at a frequency of 1007 cm^−1^. The aromatic rings’ skeletal stretching vibration and the C-O-C group of the flavonoids were both matched by the band at 1352 cm^−1^ [32]. Phenolic compounds, identified as cyanidin compounds by the absorption spectra of anthocyanins, are confirmed to be present in the microcapsules as O–H symmetric stretching vibration at 3100–3400 cm^−1^, C–H aliphatic at 2900–2840 cm^−1^, C–H aromatic at 675–870 cm^−1^, and C=C aromatic at 1660 cm^−1^ [33]. The strawberry microcapsules showed substantial anthocyanin content in all these functional groups.

### 3.4. The Effect of Encapsulation on the Thermal Degradation and Mass Loss of Anthocyanins in Strawberry Microcapsules

**Figure 5:**
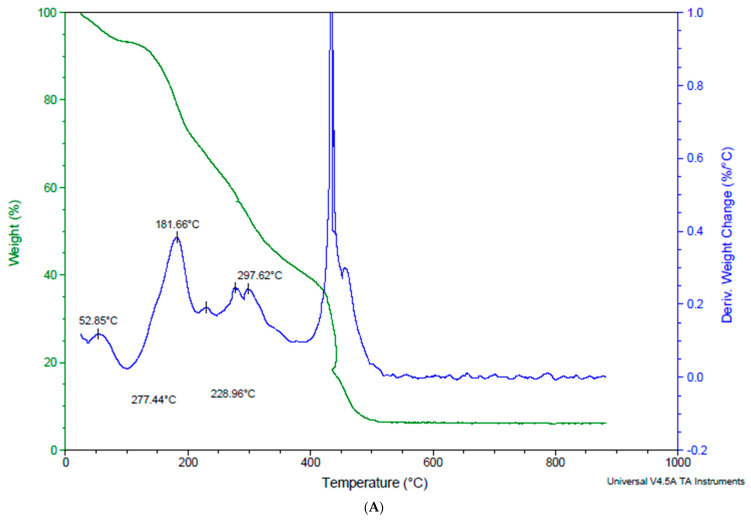

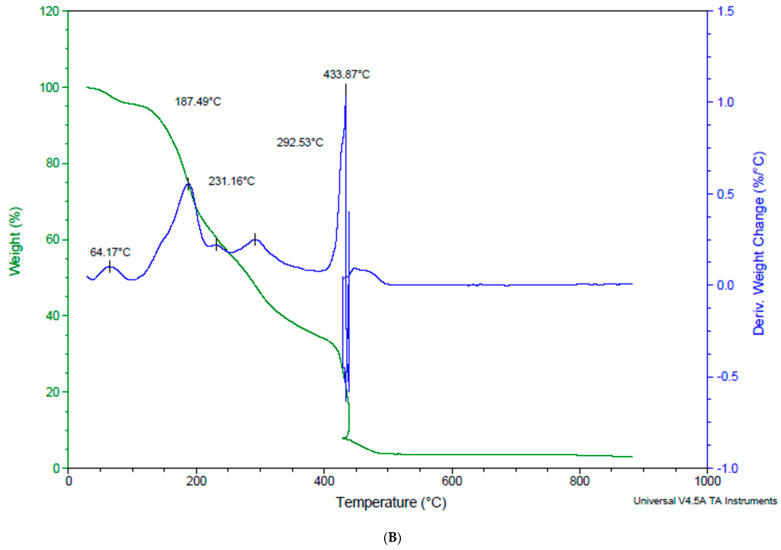

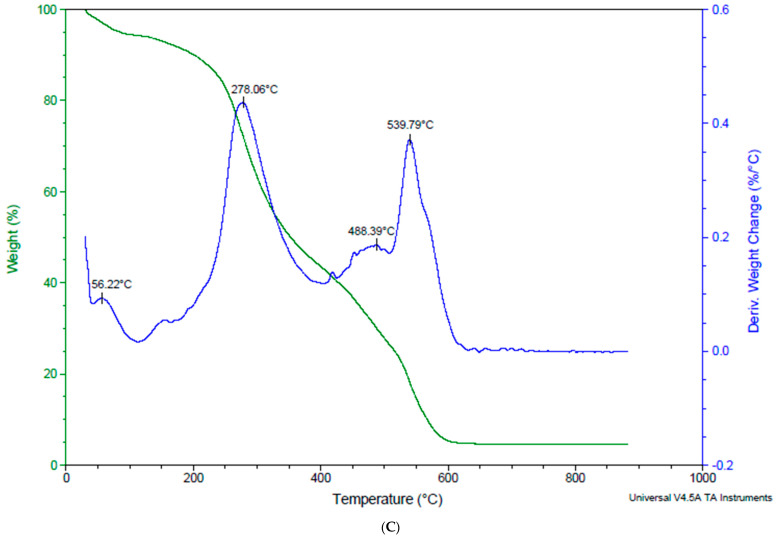

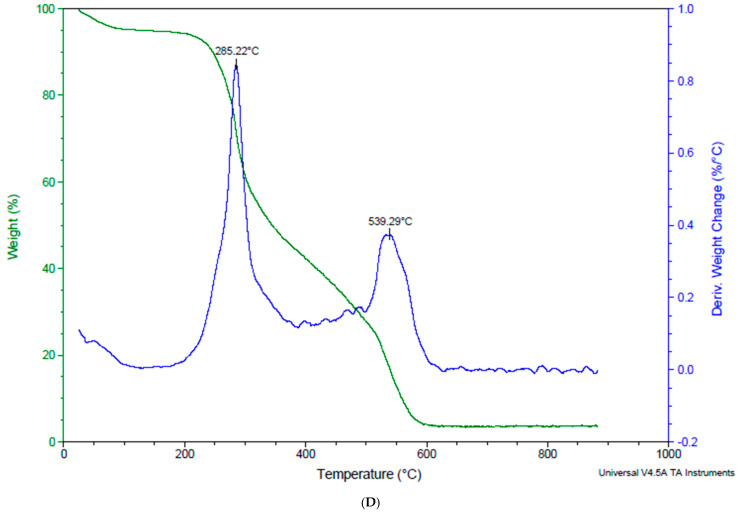
Thermal stability of strawberry microcapsules. (**A**) SJ: strawberry juice, (**B**) SJPO: strawberry (*Fragaria × ananassa*) juice + pea protein isolate + okra mucilage, (**C**) SJPP: strawberry (*Fragaria × ananassa*) juice + pea protein isolate + psyllium mucilage, (**D**) SJPPO: strawberry (*Fragaria × ananassa*) juice + pea protein isolate + okra mucilage + psyllium mucilage.

Food sensory properties, such as flavour, taste, and appearance, are impacted by anthocyanin retention. Therefore, before being included in meals, strawberry microcapsules must be verified for extended heat stability. Thermogravimetric evaluation looks at how a sample degrades thermally at a certain temperature range due to mass loss [34]. The effect of temperature on microcapsule mass loss at 10 °C and 900 °C are shown in Figure 5. The mass loss results were similar for all treatments, as reported in %/min on the mass of the derivate axis as shown in Figure 5. The evaporation of water absorbed by the microcapsules, which happens between 20 and 200 °C, is the cause of first and second mass losses. Due to the disintegration of encapsulated materials and the degradation of biopolymers, mass loss was considerable at about 280 °C. Furthermore, mass loss between 170 and 370 °C may be connected to glycosidic bond obliteration [23]. The residue amounts for the strawberry microcapsules are between 6% and 20%. Thermal analysis of organic material under nitrogen causes non-volatile structures to develop, which leads to the creation of residual carbon [35]. Ionic interactions are demonstrated by the observed variations in thermograms, which may result in the creation of new structures with a variety of thermal characteristics [35]. These results suggest that the strawberry microcapsules in this study are stable under normal physiological circumstances.

### 3.5. Effect of In Vitro Digestion on the Total Phenolic Content (TPC) and Total Anthocyanin Content (TAC) from Encapsulated Strawberry Juices

Table 1 shows the TPC and TAC of strawberry juice with different biopolymer extracts. SJPPO-encapsulated strawberry juice extracts had the highest TPC at 888.73 mg GAE/100 g DW, while SJ had the lowest at 847.25 mg GAE/100 g DW. Strawberry extract encapsulated in SJPPO had the highest TAC (84.38 mg Cy-Glu/100 g DW), while SJ had the lowest (56.86 mg Cy-Glu/100 g DW). All samples had a reduction in TPC and TAC after exposure to gastrointestinal conditions. TPC and TAC bioaccessibility of unencapsulated strawberry fruit juice (SJ) were 39.26% and 37.46%, respectively. However, using different biopolymers to encapsulate the strawberry juice improved both TPC (SJPP, 51.54%; SJPO, 48.52%; and SJPPO, 54.39%) and TAC (SJPP, 44.45%; SJPO, 41.92%; and SJPPO, 58.48%) bioaccessibility. The findings of this study are consistent with those of Seke et al. [22], who investigated the effect of encapsulating Natal plum phenolic extracts by using a combination of polysaccharides and protein isolates. In addition, Flores et al. [36] reported higher bioaccessibility of total phenol content (68%) after intestinal digestion after encapsulating blueberry extracts. Studies on engineering of delivery systems have revealed protein–polysaccharide mixes are highly effective in protecting and releasing nutraceuticals and other bioactive [27]. 

The key to effectively retaining core compounds lies in the efficient involvement of covalent bonding plus repulsion and attraction via electrostatic–electrostatic interactions with their hydrophobic compartments [22]. Encapsulation occurs due to intrinsic qualities in applied biopolymers, surfactants, and emulsifiers. The interaction types allow for the successful binding of coating agents with functional or binding sites to hydrophilic and hydrophobic bioactive compounds [10]. Furthermore, bioactive compounds can also be released or better retained as a result of interactions that are altered by the encapsulation process and environmental factors.

### 3.6. Effect of In Vitro Digestion on Anthocyanins and Phenolic Compounds from Unencapsulated and Encapsulated Strawberry Juices

Strawberry extracts contain pelargonidin 3-glucoside, cyanidin 3-glucoside, cyanidin, delphinidin, and malvidin 3-glucoside. In strawberry juice extract, pelargonidin 3-glucoside was found to be the most prevalent anthocyanin, with the highest content (49.39 ± 0.93 mg 100^−1^) in SJPPO and the least content (42.95 ± 1.74 mg 100^−1^) in unencapsulated strawberries (Table 2). During intestinal digestion, the release of anthocyanins decreased significantly, with more anthocyanins being released in non-encapsulated extracts SJ than in SJPO, SJPP, and SJPPO at the end of gastrointestinal digestion. During intestinal digestion, the release of anthocyanins decreased significantly. The bioaccessibility of the main anthocyanin pelargonidin 3-glucoside after intestinal digestion was 46.33 ± 0.55%, 49.43 ± 0.93%, 52.49 ± 0.84%, and 56.33 ± 0.73% for SJ, SJPO, SJPP, and SJPPO, respectively. Encapsulation using different biopolymers improved the bioaccessibility of anthocyanins. The medium’s pH has a major impact on the stability of anthocyanins. Although anthocyanins are stable in acidic pH environments, they experience hydration, ring fission, and the creation of ionized chalcones in neutral pH environments [13]. Commercial encapsulation appears to provide some level of protection against intestinal medium degradation but does not appear to delay the release of anthocyanins. In the comparison of three encapsulating methods, Oidtmann et al. [37] concluded that none of the systems prevented early release during simulated stomach digestion. However, compared to the commercial isolate, the enclosed systems induced higher ultimate anthocyanin release (23%). McDougall et al. [38] investigated how different food matrixes affected raspberry anthocyanin digestion and found that phenols momentarily bonded to those matrixes and delayed their decomposition. Protein-based wall materials were found to sustain antioxidant activity better than carbohydrate-rich encapsulants [39]. Encapsulation requires less protein-based components than polysaccharides in terms of mass [40] and ensures that anthocyanins reach the gastrointestinal tract without degradation, thus protecting them for optimal performance.

Ellagic acid, chlorogenic acid, catechin, and kaempferol were the most abundant phenolic compounds in the undigested strawberry juice powder samples, at 156.74 ± 0.85 mg GAE/100 g, 148.62 ± 0.62 mg GAE/100 g, 78.33 ± 1.43 mg GAE/100 g, and 69.57 ± 0.43 mg GAE/100 g, respectively (Table 2). Milosavljević et al. [2] reported these compounds represent approximately 85% of total phenolics, apart from anthocyanins. Among strawberry flavonoids are catechin, quercetin, and kaempferol derivatives which are typical of the Rosaceae family’s red or yellowish berry fruit members [41]. Phenolic acid composition during digestion might vary according to research based on food matrix composition [42]. In addition to their original forms, these molecules can undergo transformation or degradation into chemical groups that possess unique characteristics [42]. The composition of bioactive components in target organs may be affected by various mechanisms, such as liver metabolism and excretion, for which pharmacokinetic aspects and metabolic interactions must be assessed [5]. Phenolic bioaccessibility is influenced by pH exposure, compound structure, and fruit matrix composition. The overall trend of phenolic compounds in all samples decreased significantly during digestion. Furthermore, Kosiska-Cagnazzo et al. [43] reported that strawberry phenolic acid composition and anthocyanin composition changed profoundly in vitro. The loss of phenolic acids could be caused by pH 7 and bile salts [44]. The bioaccessibility of the encapsulated and unencapsulated strawberry juice phenolic compounds ranged between 43.90% and 48.40% for catechin, 28.59% and 42.59% for chlorogenic acid, 33.52% and 63.25% for epicatechin, 33.20% and 73.72% for ellagic acid, and 16.13% and 40.47% for kaempferol (Table 2). SJPPO had the highest bioaccessibility for individual phenolic compounds, suggesting that the wall material reduced phytochemical losses in the stomach. It is possible that they formed aggregates in the presence of acid. It is also possible that some phytochemicals slowly leak out of the microcapsules as they dissolve in the neutral-alkaline solution of the intestinal fluid, resulting in an increase in phytochemical concentration in the intestinal phase compared to unencapsulated extracts. According to Cervantes et al. [45], strawberry phenolic compounds including ellagic, chlorogenic, and procyanidin B1 acids were the most bioavailable at the intestinal level. Dietary polyphenols have been demonstrated to help maintain human health, particularly in the gut, by encouraging beneficial bacteria growth while restraining pathogenic bacteria growth, creating a prebiotic-like effect [46]. Ingestion of polyphenols does not reach the colon most of the time because they are poorly absorbed and metabolized by human enzymes such as lipase and β-glucosidae during their passage through the upper gastrointestinal tract. The results of this study showed that encapsulation may improve the survival of polyphenols in gastrointestinal digestion conditions, possibly making them bioavailable in the colon.

### 3.7. In Vitro Antioxidant Capacity of Unencapsulated and Encapsulated Strawberry (Fragaria × ananassa) Juices

**Figure 6:**
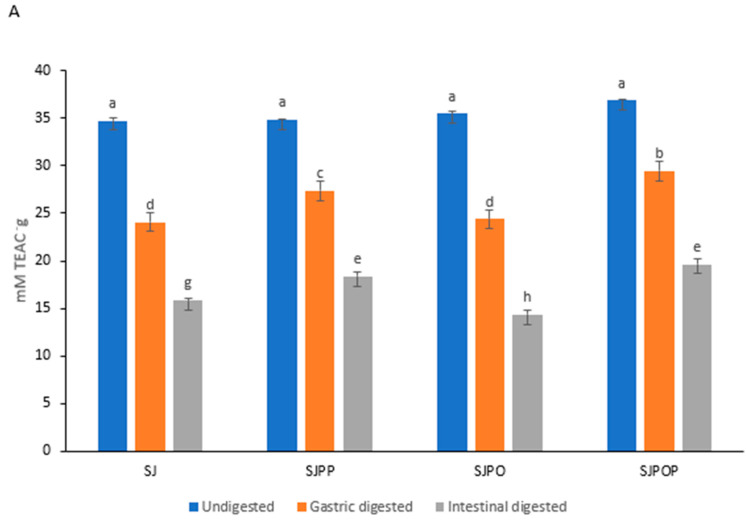

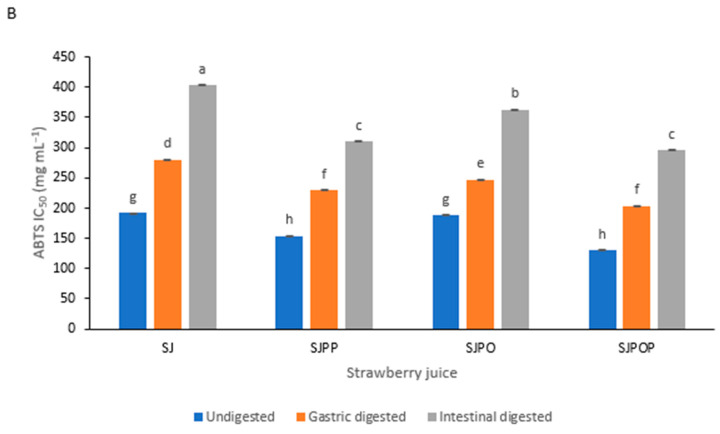

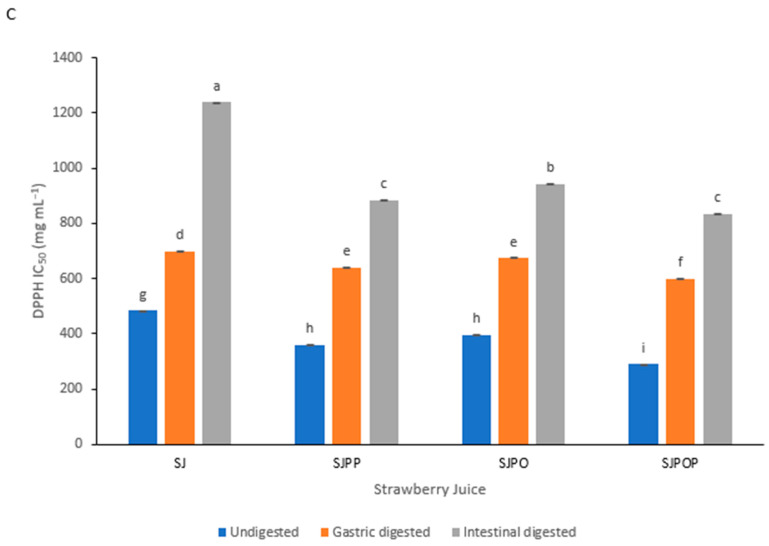
Effect of encapsulation of strawberry (*Fragaria × ananassa*) juice on FRAP (**A**), ABTS (**B**), and DPPH (**C**) scavenging activity. Error bars show the standard deviation value, bars with different letters are significantly different *p* < 0.05. SJ: strawberry (*Fragaria × ananassa*) juice, SJPP: strawberry (*Fragaria × ananassa*) juice + pea protein isolate + psyllium mucilage, SJPO: strawberry (*Fragaria × ananassa*) juice + pea protein isolate + okra mucilage, SJPPO: strawberry (*Fragaria × ananassa*) juice + pea protein isolate + okra mucilage + psyllium mucilage.

DPPH, FRAP, and ABTS were used to assess the antioxidant activity of undigested and digested (encapsulated or unencapsulated) strawberry juice extract. The results are shown as an IC_50_ value. Figure 6 illustrates how the antioxidant activity of the unencapsulated and encapsulated strawberry juice extracts changed throughout in vitro digestion. DPPH scavenging activity, ABTS capacity, and FRAP activity decreased as in vitro digestion progressed in all treatments. A lower IC_50_ value indicates more significant antioxidant activity. This study obtained the highest antioxidant activity (the lowest IC50 values) for SJPPO-encapsulated strawberry juice before digestion (undigested) and in the gastric phase. However, SJPPO- and SJPO-encapsulated strawberry juice showed the highest antioxidant scavenging activity in the intestinal phase; this can be attributed to the phenolic and anthocyanin composition in the intestinal phase. The ABTS scavenging activity also showed similar trends, showing that SJPPO- and SJPO-encapsulated strawberry juice showed the highest antioxidant scavenging activity in the intestinal phase. In contrast, SJPPO-encapsulated strawberry juice had the highest antioxidant power (FRAP) in the gastric phase relative to the other encapsulations. However, SJPPO- and SJPO-encapsulated strawberry juice exhibited the highest antioxidant power in the intestinal phase. The concentration of phenolic compounds in the intestinal phase was lower than in the undigested freeze-dried strawberry samples, resulting in a decrease in antioxidant activity. pH changes and digestive enzymes may significantly influence the susceptibility of this sensitive category of substances during digestion. As a result, there is a reduction in polyphenol stability and antioxidant activity. The susceptibility of polyphenols to neutral pH or polyphenol bioconversion to other structural types with distinct chemical characteristics may also be the cause of the decline in FRAP and DPPH levels [47]. However, the antioxidant activity of the strawberry juice extract was preserved during the encapsulating process. Comparatively to unencapsulated samples, encapsulated samples displayed the highest antioxidant activity values in the intestinal phase. A significant reason for this is the insulating properties of the wall material used in the encapsulation [22]. In addition, electrons from the barrier layer can participate in the antioxidant process, which increases the antioxidant effects of encapsulated fruit juices [9]. The redox characteristics of phenols allow them to operate as radical scavengers, hydrogen donors, and singlet oxygen quenchers, contributing to their antioxidant activity. Additionally, the amount and location of hydrogen-donating hydroxyl groups on the aromatic ring of phenol molecules affect free radical scavenging and antioxidant ability [48]. Also, releasing amino acids or sugars from the original dietary components may raise the overall antioxidant capacity assay readings.

A strong correlation was determined between FRAP and TPC (R = 0.93), TAC (R = 0.79), ellagic acid (R = 0.83), catechin (R = 0.73), chlorogenic acid (0.69), pelargonidin 3-glucoside (R = 0.53), cyanidin 3-glucoside (R = 0.86), cyanidin (R = 0.71), delphinidin (R = 0.62), and malvidin 3-glucoside (R = 0.74) (Supplementary Table S3). Similar strong correlations were previously observed between FRAP and TPC, and FRAP and TAC, by Seke et al. (2022). The ABTS radical scavenging showed a strong positive correlation with TPC (0.79), TAC (0.82), ellagic acid (0.78), chlorogenic acid (0.82), cyanidin 3-glucoside (R = 0.72), and malvidin 3-glucoside (R = 0.85). The DPPH radical scavenging activity showed a positive correlation with TPC (0.85), TAC (0.82), ellagic acid (R = 0.81), chlorogenic acid (R = 0.75), delphinidin (R = 0.57), cyanidin (R = 0.83), pelargonidin 3-glucoside (R = 0.53), and malvidin 3-glucoside (R = 0.85). These findings imply that the phenolic components of strawberry juice are mainly responsible for its antioxidant qualities. Because of their chemical makeup, phenols have a major effect on antioxidant activity. It has been found that the carboxyl (COOH) grouping of hydroxybenzoic acids is less capable of protonation and radical stabilization than the CHCHCOOH clustering of hydroxycinnamic acids [48]. Flavonoids alone have the ability to donate electrons or protons, depending on their structure, resulting in a strong bond.

## 4. Conclusions

Strawberry microcapsules have high antioxidant capacity, TPC, TAC, and thermal stability. The potent red colour attributed to the presence of anthocyanins possibly allows strawberry microcapsules to be used as food colourants. SEM morphologies showed an amorphous structure in SJ, SJPO, SJPP, and SJPPO, and this was also confirmed by x-ray diffraction analysis, which further indicated a tighter crosslinking density in SJPPO. The bioaccessibility of anthocyanins and phenolic compounds was also improved through the use of encapsulation using multiple biopolymers as wall materials. The bioaccessibility of the main anthocyanin pelargonidin 3-glucoside after intestinal digestion was 46.33 ± 0.55% for SJ, improved to 49.43 ± 0.93%, 52.49 ± 0.84%, and 56.33 ± 0.73% for SJPO, SJPP, and SJPPO, respectively. A strong correlation was observed between FRAP, DPPH, and ABTS and TPC, TAC, individual phenolic compounds, and anthocyanins, with R values ranging between 0.53 and 0.93. Encapsulation can improve anthocyanin stability, enabling the possible use of strawberry byproducts in the food sector as functional ingredients.

## Figures and Tables

**Table 1:** An evaluation of the effect of simulated in vitro digestion on the total phenolic and total anthocyanin content of strawberry (*Fragaria × ananassa*) juice extracts.

	Undigested	Gastric Digested	Intestinal Digested	Bioaccessibility (%)
TAC (mg Cy-Glu/100 g)				
SJ	56.86 ^a^ ± 0.55	32.73 ^b^ ± 0.39	21.30 ^c^ ± 0.12	37.46 ^d^ ± 0.23
SJPP	72.58 ^a^ ± 0.70	56.47 ^b^ ± 0.26	32.28 ^c^ ± 0.41	44.45 ^b^ ± 0.47
SJPO	68.71 ^a^ ± 0.63	47.01 ^b^ ± 0.59	28.81 ^c^ ± 0.16	41.92 ^c^ ± 0.83
SJPPO	84.38 ^a^ ± 0.82	63.72 ^b^ ± 0.44	49.35 ^c^ ± 0.33	58.48 ^a^ ± 0.61
TPC (mg GAE/100 g)				
SJ	847.25 ^a^ ± 5.36	568.75 ^b^ ± 1.75	332.71 ^c^ ± 3.15	39.26 ^d^ ± 0.93
SJPP	875.88 ^a^ ± 4.28	721.96 ^b^ ± 3.38	451.39 ^c^ ± 2.17	51.54 ^b^ ± 0.84
SJPO	869.52 ^a ±^ 3.76	688.63 ^b^ ± 2.97	421.71 ^c^ ± 2.02	48.52 ^c^ ± 0.83

The means in a row that are separated by the same letter ± standard deviation do not vary statistically at *p* < 0.05. Within the bioaccessibility column, means denoted by the same letter ± standard deviation show no significant difference at *p* < 0.05. SJ: strawberry juice, SJPO: strawberry (*Fragaria × ananassa*) juice + pea protein isolate + okra mucilage, SJPP: strawberry (*Fragaria × ananassa*) juice + pea protein isolate + psyllium mucilage, SJPPO: strawberry (*Fragaria × ananassa*) juice + pea protein isolate + okra mucilage + psyllium mucilage, TPC: total phenolic content, TAC: total anthocyanin content.

**Table 2:** Changes in anthocyanin and phenolic components in strawberry (*Fragaria × ananassa*) juice samples during gastrointestinal digestion on a dry weight basis.

	Undigested	Gastric Digested	Intestinal Digested	Bioaccessibility (%)
SJ				
Anthocyanins (mg/100 g)				
Pelargonidin 3-glucoside	33.65 ^a^ ± 2.38	25.28 ^b^ ± 1.04	10.82 ^c^ ± 0.84	32.15 ^j^ ± 0.92
Cyanidin 3-glucoside	37.05 ^a^ ± 0.50	28.40 ^b^ ± 0.70	15.49 ^c^ ± 0.30	41.81 ^f^ ± 0.75
Cyanidin	28.50 ^a^ ± 0.17	18.35 ^b^ ± 0.28	9.89 ^c^ ± 0.81	34.70 ^I^ ± 0.28
Delphinidin	26.34 ^a^ ± 0.23	15.78 ^b^ ± 0.19	8.43 ^c^ ± 0.64	32.00 ^j^ ± 0.62
Malvidin 3-glucoside	42.95 ^a^ ± 1.74	33.90 ^b^ ± 0.59	20.04 ^c^ ± 0.62	46.65 ^e^ ± 0.55
SJPPO				
Pelargonidin 3-glucoside	55.83 ^a^ ± 1.08	34.91 ^b^ ± 1.41	25.22 ^c^ ± 0.75	45.17 ^e^ ± 0.92
Cyanidin 3-glucoside	48.67 ^a^ ± 0.82	32.49 ^b^ ± 0.48	28.52 ^c^ ± 0.59	58.60 ^a^ ± 0.39
Cyanidin	37.81 ^a^ ± 0.39	25.53 ^b^ ± 0.80	19.89 ^c^ ± 0.81	52.61 ^c^ ± 0.70
Delphinidin	34.39 ^a^ ± 0.15	19.37 ^b^ ± 0.39	17.01 ^c^ ± 0.44	49.46 ^d^ ± 1.03
Malvidin 3-glucoside	49.39 ^a^ ± 0.93	36.68 ^b^ ± 0.37	27.82 ^c^ ± 0.53	56.33 ^b^ ± 0.73
SJPO				
Pelargonidin 3-glucoside	38.18 ^a^ ± 1.81	22.34 ^b^ ± 0.91	14.21 ^c^ ± 0.87	37.22 ^h^ ± 0.54
Cyanidin 3-glucoside	37.51 ^a^ ± 0.98	24.40 ^b^ ± 0.64	18.59 ^c^ ± 0.71	49.56 ^d^ ± 0.82
Cyanidin	28.08 ^a^ ± 0.67	17.57 ^b^ ± 0.85	12.19 ^c^ ± 0.21	43.41 ^f^ ± 1.03
Delphinidin	26.08 ^a^ ± 0.78	16.39 ^b^ ± 0.28	10.34 ^c^ ± 0.40	39.65 ^g^ ± 0.46
Malvidin 3-glucoside	40.76 ^a^ ± 1.28	29.49 ^b^ ± 0.55	20.15 ^c^ ± 0.27	49.43 ^d^ ± 0.93
SJPP				
Pelargonidin 3-glucoside	36.84 ^a^ ± 1.03	27.33 ^b^ ± 0.94	19.64 ^c^ ± 0.81	53.31 ^c^ ± 0.43
Cyanidin 3-glucoside	40.52 ^a^ ± 0.38	31.84 ^b^ ± 0.72	20.93 ^c^ ± 0.87	51.65 ^c^ ± 0.95
Cyanidin	30.48 ^a^ ± 0.63	19.53 ^b^ ± 0.92	13.87 ^c^ ± 0.17	45.51 ^e^ ± 0.28
Delphinidin	28.51 ^a^ ± 0.27	17.53 ^b^ ± 0.38	10.87 ^c^ ± 0.29	38.13 ^g^ ± 0.41
Malvidin 3-glucoside	45.27 ^a^ ± 0.97	36.65 ^b^ ± 0.85	23.76 ^c^ ± 0.38	52.49 ^c^ ± 0.84
Phenolic Content (mg GAE/100 g)				
Catechin	78.33 ^a^ ± 1.43	41.91 ^b^ ± 0.78	34.39 ^c^ ± 0.10	43.90 ^g^ ± 0.81
Chlorogenic acid	148.62 ^a^ ± 0.72	88.37 ^b^ ± 0.09	42.50 ^c^ ± 0.13	28.59 ^k^ ± 0.37
Ellagic acid	156.74 ^a^ ± 0.85	67.18 ^c^ ± 0.30	72.04 ^b^ ± 0.19	45.96 ^f^ ± 0.49
Kaempferol	69.51 ^a^ ± 0.43	24.82 ^b^ ± 0.29	11.21 ^c^ ± 0.44	16.13 ^l^ ± 0.75
SJPP				
Catechin	82.38 ^a^ ± 0.93	51.43 ^b^ ± 0.46	38.78 ^c^ ± 0.64	47.07 ^e^ ± 0.55
Chlorogenic acid	154.82 ^a^ ± 0.58	84.91 ^b^ ± 0.91	64.65 ^c^ ± 0.33	41.76 ^h^ ± 0.72
Epicatechin	64.87 ^a^ ± 0.38	47.33 ^b^ ± 0.27	36.12 ^c^ ± 0.43	55.68 ^c^ ± 0.94
Ellagic acid	160.41 ^a^ ± 0.54	79.37 ^c^ ± 0.83	84.71 ^d^ ± 0.29	52.81 ^d^ ± 1.03
Kaempferol	77.23 ^a^ ± 0.92	35.41 ^b^ ± 0.25	27.32 ^c^ ± 0.07	35.38 ^i^ ± 0.95
SJPO				
Catechin	80.17 ^a^ ± 0.31	49.39 ^b^ ± 1.08	36.39 ^c^ ± 0.10	45.39 ^f^ ± 0.72
Chlorogenic acid	149.74 ^a^ ± 0.71	70.65 ^b^ ± 0.97	52.48 ^c^ ± 0.62	35.05 ^i^ ± 0.69
Epicatechin	60.23 ^a^ ± 0.45	43.03 ^b^ ± 0.82	26.91 ^c^ ± 0.14	44.68 ^f^ ± 0.44
Ellagic acid	156.38 ^a^ ± 0.56	74.82 ^c^ ± 0.74	82.27 ^b^ ± 0.53	52.60 ^d^ ± 0.23
Kaempferol	64.19 ^a^ ± 0.38	32.07 ^b^ ± 0.45	20.32 ^c^ ± 0.14	31.65 ^j^ ± 0.82
SJPPO				
Catechin	86.59 ^a^ ± 2.03	57.79 ^b^ ± 0.81	41.91 ^c^ ± 0.75	48.40 ^e^ ± 0.53
Chlorogenic acid	169.23 ^a^ ± 0.72	90.72 ^b^ ± 0.94	72.08 ^c^ ± 0.28	42.59 ^g^ ± 0.82
Epicatechin	77.75 ^a^ ± 0.58	59.67 ^b^ ± 0.72	49.18 ^c^ ± 0.90	63.25 ^b^ ± 0.29
Ellagic acid	167.40 ^a^ ± 0.50	98.83 ^c^ ± 0.95	123.42 ^b^ ± 0.97	73.72 ^a^ ± 0.48
Kaempferol	84.67 ^a^ ± 0.22	59.41 ^b^ ± 0.98	34.25 ^c^ ± 0.79	40.47 ^h^ ± 0.39

The means in a row that are separated by the same letter ± standard deviation do not vary statistically at *p* < 0.05. Within the bioaccessibility column, means denoted by the same letter ± standard deviation show no significant difference at *p* < 0.05. SJ: strawberry juice, SJPO: strawberry (*Fragaria × ananassa*) juice + pea protein isolate + okra mucilage, SJPP: strawberry (*Fragaria × ananassa*) juice + pea protein isolate + psyllium mucilage, SJPPO: strawberry (*Fragaria × ananassa*) juice + pea protein isolate + okra mucilage + psyllium mucilage.

## Data Availability

Data are available upon request.

## Supplementary Materials

The following supporting information can be downloaded at: https://www.mdpi.com/article/10.3390/foods12214045/s1. Supplementary Table S1: Sample formulations; Supplementary Table S2: Phenolic and anthocyanin identification and quantification using HPLC-DAD; Supplementary Table S3: Correlation between antioxidant activities and polyphenols; Supplementary Figure S1: In vitro digestion flow diagram; Supplementary Figure S2: Chromatograms showing the effect of in vitro digestion on selected strawberry microcapsules.
