# Supplementary material for: In Vitro Antioxidant Activity, Bioaccessibility, and Thermal Stability of Encapsulated Strawberry Fruit (Fragaria × ananassa) Polyphenols
Source: Foods. 2023 Nov 6;12(21):4045. doi: 10.3390/foods12214045 (PMC10647287; doi:10.3390/foods12214045)
Supplement: Supplementary file 1 [file foods-12-04045-s001.zip › foods-2669258-supplementary.pdf]

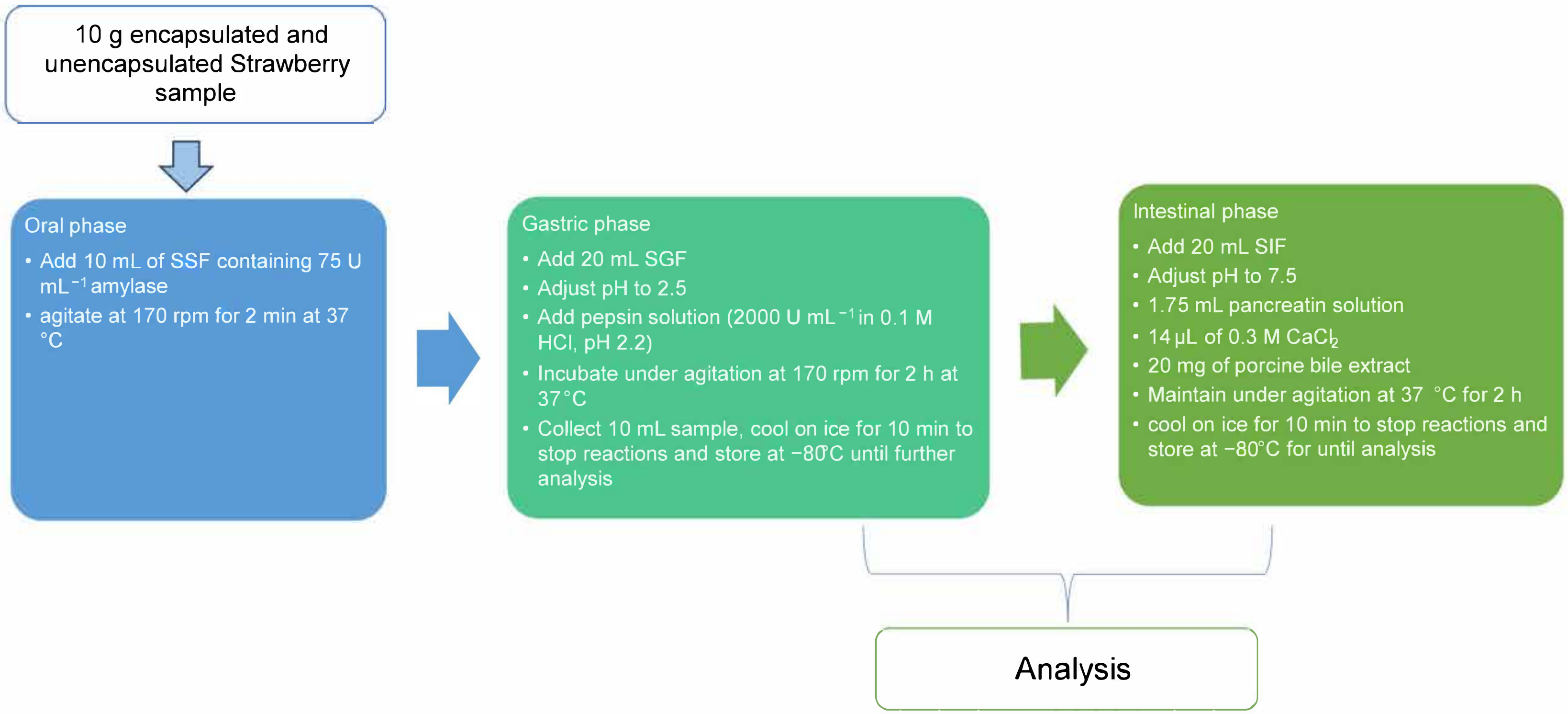

Supplementary Figure S1: *In vitro* digestion flow diagram.
SSF: simulated salivary fluid; SGF: simulated gastric fluid; SIF: simulated intestinal fluid.

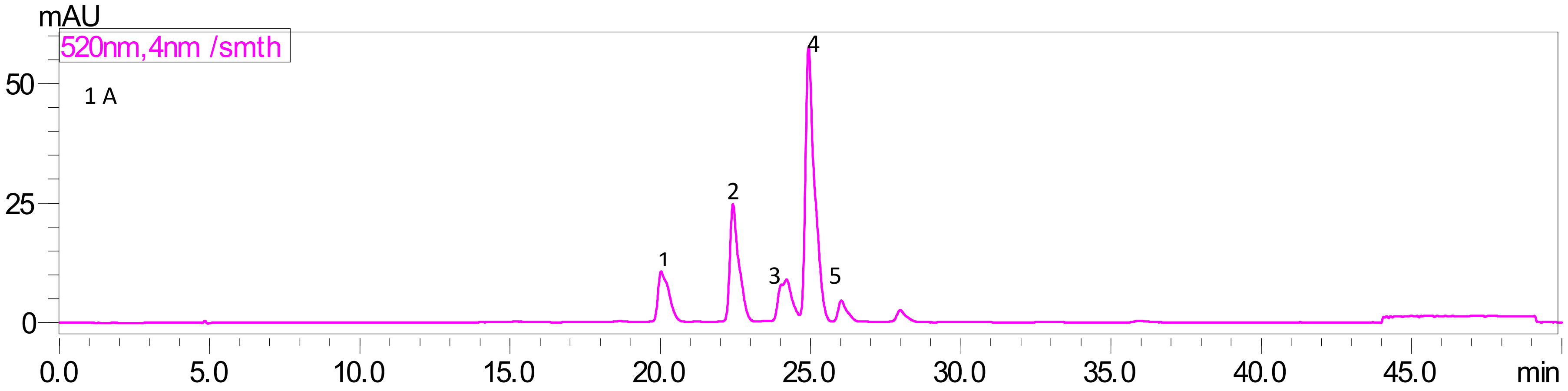

mAU
520nm,4nm /smth
1 A
50
25
0
1
2
3
4
5
0.0
5.0
10.0
15.0
20.0
25.0
30.0
35.0
40.0
45.0
min

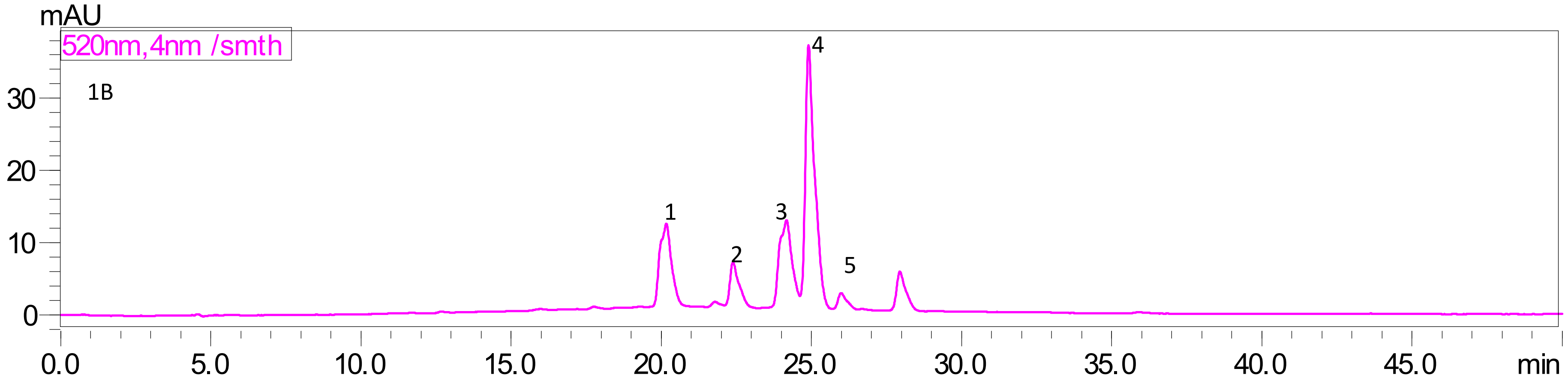

mAU
520nm,4nm /smth
1B
30
20
10
0
1
2
3
4
5
0.0
5.0
10.0
15.0
20.0
25.0
30.0
35.0
40.0
45.0
min

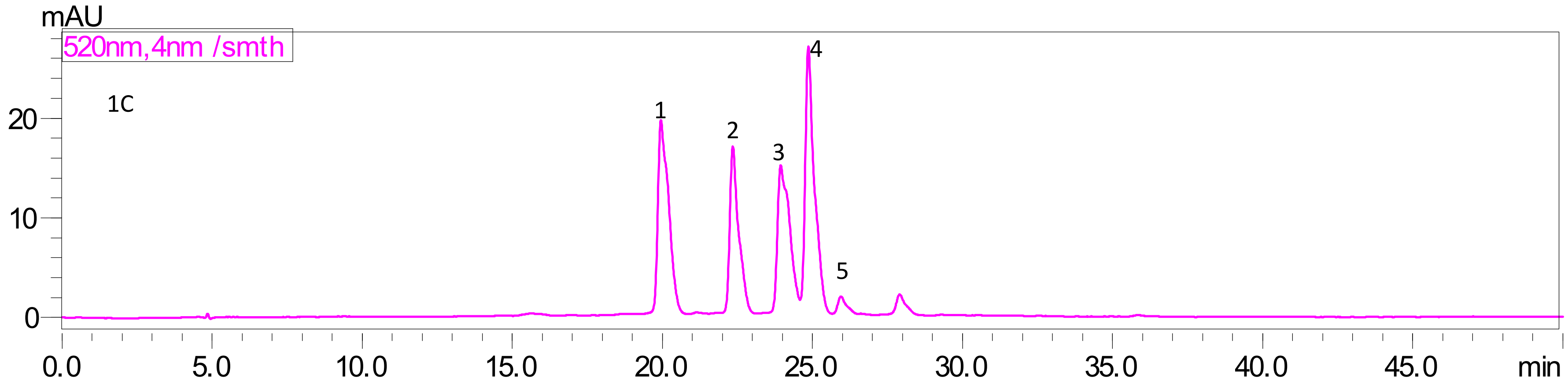
mAU
520nm,4nm /smth
1C
20
10
0
1
2
3
4
5
0.0
5.0
10.0
15.0
20.0
25.0
30.0
35.0
40.0
45.0
min

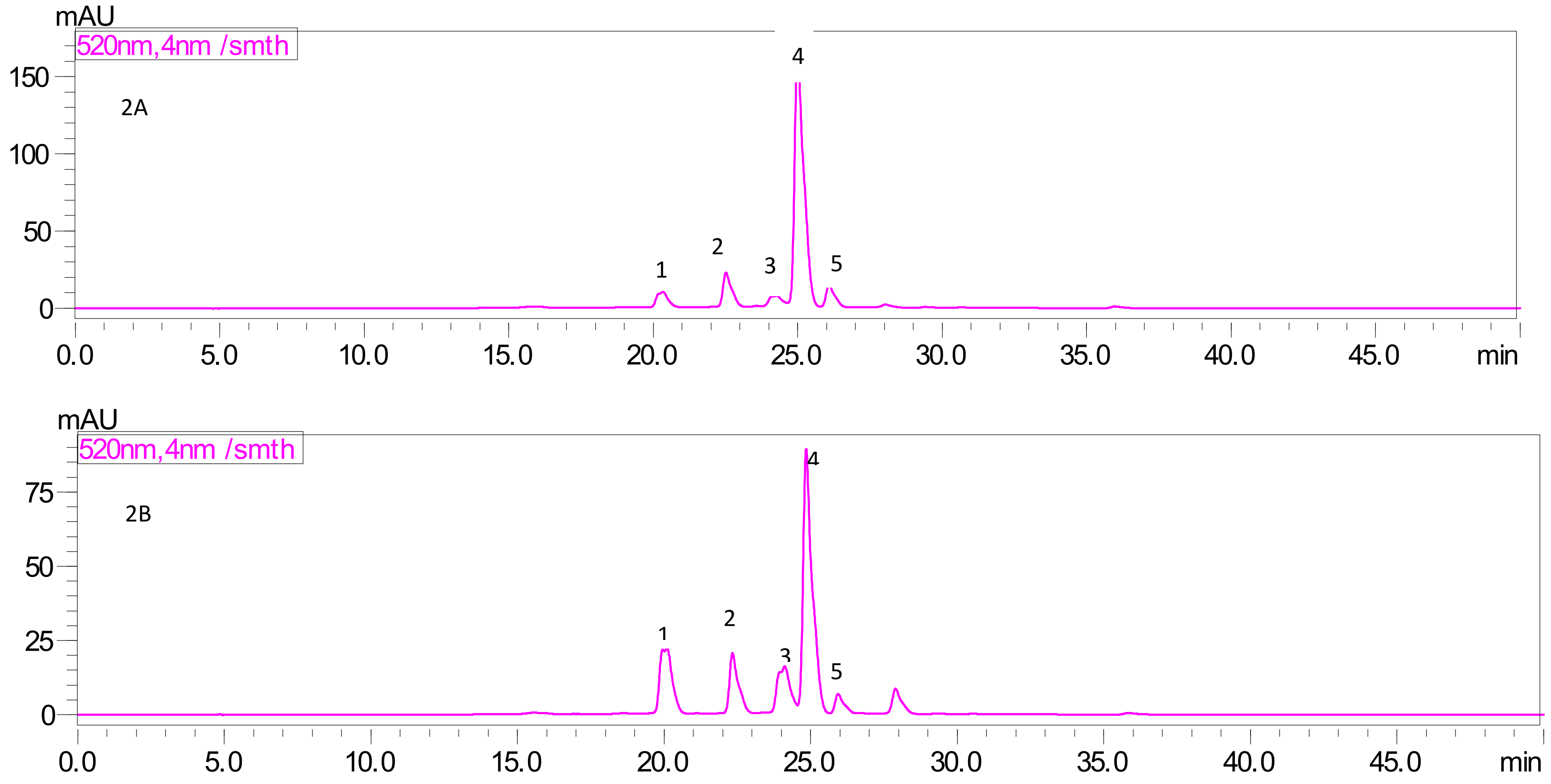
mAU
520nm,4nm /smth
2A
150
100
50
0
1
2
3
4
5
0.0
5.0
10.0
15.0
20.0
25.0
30.0
35.0
40.0
45.0
min
mAU
520nm,4nm /smth
2B
75
50
25
0
1
2
3
4
5
0.0
5.0
10.0
15.0
20.0
25.0
30.0
35.0
40.0
45.0
min

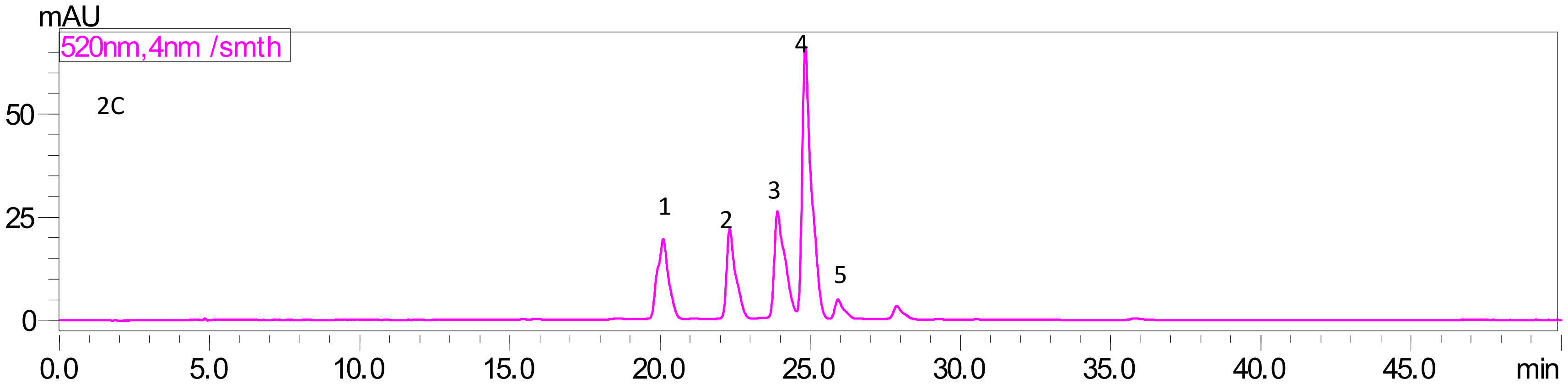

Supplementary Figure S2: Chromatograms showing the effect of *in vitro* digestion on selected strawberry microcapsules. 1A: SJ undigested; 1B: SJ gastric digested; 1C: SJ intestinal digested: 2A: SJPPO undigested; 2B: SJPPO gastric digested; 2C: SJPPO intestinal digested. 1: malvidin-3-glucoside, 2: cyanidin-3-glucoside, 3: cyanidin, 4: Pelargonidin-3-glucoside and 5: delphinidin

Supplementary Table S1: Sample formulations

		Code
1.	strawberry (*Fragaria x ananassa*) juice	SJ
2.	strawberry (*Fragaria x ananassa*) juice + pea protein isolate+ psyllium mucilage	SJPP
3.	strawberry (*Fragaria x ananassa*) juice + pea protein isolate+ okra mucilage	SJPO
4.	strawberry (*Fragaria x ananassa*) juice + pea protein isolate+ okra mucilage+ psyllium mucilage	SJPOP

Supplementary Table S2: Phenolic and anthocyanin identification and quantification using HPLC-

Phenolic	Retention time (min)	Regression equation	R^2	LOD (μg/L)	LOQ (μg/L)
Catechin	13.24	y=2828x-69172	0.999	3.2	15.7
Ellagic acid	14.2	y=35316x+193517	0.997	1.4	3.3
Chlorogenic	14.724	y=71930x-2110,5	0.995	0.11	0.37
Kaempferol	16.448	y=26658x+492185	0.996	2.7	9.3
Pelargonidin-3-glucoside	25.746	y=20110x+9484.9	0.997	0.31	1.2
Cyanidin-3-glucoside	22.591	y=75813x-107617	0.999	0.05	0.18
Cyanidin	24.819	y=6925.3x-62646	0.999	10.25	34.17
Delphinidin	26.382	y=19722x-341718	0.998	3.2	10.9
Malvidin-3-glucoside	20.957	y=20067x-279209	0.999	3.3	11.9

Supplementary Table S3: Correlation between antioxidant activities and polyphenols

	**ABTS (R-value)**	**DPPH (R-value)**	**FRAP (R-value)**
TA	0.82	0.82	0.79
Pelargonidin-3-glucoside	0.69	0.53	0.53
Cyanidin-3-glucoside	0.72	0.83	0.86
Cyanidin	0.47	0.69	0.71
Delphinidin	0.52	0.57	0.61
Malvidin-3-glucoside	0.74	0.85	0.74
Catechin	0.80	0.49	0.73
Chlorogenic acid	0.82	0.75	0.69
Ellagic acid	0.72	0.81	0.83
Kaempferol	0.35	0.33	0.42
TPC	0.79	0.79	0.93
